# Supplementation with milk enriched with complex lipids during pregnancy: A double-blind randomized controlled trial

**DOI:** 10.1371/journal.pone.0244916

**Published:** 2021-02-24

**Authors:** Benjamin B. Albert, José G. B. Derraik, Yin-Yin Xia, Tom Norris, Ting Zhang, Ting-Li Han, Chen Chang, Angela Rowan, Sophie Gallier, Renato T. Souza, Judith J. Hammond, Wei Zhou, Hua Zhang, Hong-Bo Qi, Philip N. Baker

**Affiliations:** 1 Liggins Institute, University of Auckland, Auckland, New Zealand; 2 Department of Women’s and Children’s Health, Uppsala University, Uppsala, Sweden; 3 School of Public Health and Management, Research Center for Medicine and Social Development, Innovation Center for Social Risk Governance in Health, Chongqing Medical University, Chongqing, China; 4 College of Life Sciences, University of Leicester, Leicester, United Kingdom; 5 Department of Obstetrics and Gynaecology, The First Affiliated Hospital of Chongqing Medical University, Chongqing, China; 6 Canada-China-New Zealand Joint Laboratory of Maternal and Fetal Medicine, Chongqing Medical University, Chongqing, China; 7 Fonterra Co-operative Group Ltd, Palmerston North, New Zealand; 8 Department of Obstetrics and Gynaecology, University of Campinas, Campinas, Brazil; 9 Auckland UniServices Ltd, Auckland, New Zealand; 10 Department of Obstetrics, Chongqing Health Centre for Women and Children, Chongqing, China; University of Ghana, GHANA

## Abstract

**Background:**

Gangliosides are a class of sphingolipids that are present in the cell membranes of vertebrates. Gangliosides influence a broad range of cellular processes through effects on signal transduction, being found abundantly in the brain, and having a role in neurodevelopment.

**Objective:**

We aimed to assess the effects of maternal daily consumption of ganglioside-enriched milk vs non-enriched milk and a non-supplemented group of pregnant women on maternal ganglioside levels and pregnancy outcomes.

**Design:**

Double-blind parallel randomized controlled trial.

**Methods:**

1,500 women aged 20–40 years were recruited in Chongqing (China) between 11 and 14 weeks of a singleton pregnancy, and randomized into three groups: Control–received standard powdered milk formulation (≥4 mg gangliosides/day); Complex milk lipid-enhanced (CML-E) group–same formulation enriched with complex milk lipids (≥8 mg gangliosides/day) from milk fat globule membrane; Reference–received no milk. Serum ganglioside levels were measured in a randomly selected subsample of 250 women per group.

**Results:**

CML-E milk was associated with marginally greater total gangliosides levels in maternal serum compared to Control (13.02 vs 12.69 μg/ml; p = 0.034) but not to Reference group. CML-E milk did not affect cord blood ganglioside levels. Among the 1500 women, CML-E milk consumption was associated with a lower rate of gestational diabetes mellitus than control milk [relative risk 0.80 (95% CI 0.64, 0.99)], but which was not different to the Reference group. CML-E milk supplementation had no other effects on maternal or newborn health.

**Conclusions:**

Maternal supplementation with milk fat globule membrane, as a source of gangliosides, was not associated with any adverse health outcomes, and did not increase serum gangliosides compared with the non-supplemented reference group.

**Trial registration:**

Chinese Clinical Trial Register (ChiCTR-IOR-16007700).

**Clinical trial registration:**

ChiCTR-IOR-16007700; www.chictr.org.cn/showprojen.aspx?proj=12972.

## Introduction

Gangliosides are a class of sialic acid containing glycosphingolipids found in almost all vertebrate cell membranes [1]. They have an essential role in brain structure and function [2,3], and are found most abundantly in the central nervous system [4]. Gangliosides influence a broad range of cellular processes through their effects on signal transduction (including cellular signalling in immune cells and neurons), and play an important role in neural development [2,3]. Thus, modulating ganglioside levels through dietary intervention could have a range of biological effects. Due to their high content in the brain, the effects of gangliosides on brain development [5–8] and cognition [9–15] have received most interest.

There is rapid accretion of complex lipids (such as gangliosides and phospholipids) into neuronal tissues *in utero* and during neonatal life [16,17]. Dietary ganglioside consumption increases plasma concentrations [18], although the effects of associated dietary factors such as micronutrients on absorption are unknown. Limited evidence demonstrates that at least some ganglioside species are transferred across the placenta. In the rat, a radioisotope study showed that the ganglioside GM1 crossed the placenta [19]. GM3 also crossed human placenta but for GD3, the dominant ganglioside found in milk, it was unclear whether it crossed or was metabolized by the placenta [20]. A rodent model has shown that increasing maternal intake does lead to greater levels of gangliosides in the fetal brain [21]. However, evidence in humans is missing.

Dietary sources of gangliosides are animal-based foods, such as meat, eggs, and full-fat dairy products [22]. In human and bovine milks, lipids are stabilised within the milk fat globule membrane (MFGM), which is rich in complex milk lipids (CMLs) including phospholipids and gangliosides [23–26]. Thus, food products enriched with MFGM could be used as dietary supplements during pregnancy to enhance the levels of gangliosides both in the mother and the developing fetus.

The effects of dietary supplementation using MFGM (as a source of gangliosides and phospholipids) during pregnancy have not been investigated. However, supplementation of formula-fed infants with a MFGM-rich ingredient improved cognitive performance in the first year of life [14,15], supporting the results of previous animal studies [12,13]. Supplementation during pregnancy may be efficacious, as the accrual of complex lipids in neuronal tissue starts early *in utero* and is rapid in fetal life [16,17]. However, as gangliosides influence a broad range of cellular processes, it is critical that the effects of prenatal supplementation on pregnancy and neonatal outcomes are determined.

Therefore, we performed a large three-group, double-blind parallel randomised controlled trial comparing the effects of daily consumption of a CML-enriched milk product with a control milk product and a reference group of pregnant women on maternal ganglioside levels and pregnancy outcomes.

## Materials and methods

Ethics approval for this study was provided by The Ethics Committee of ChongQing Medical University (#2014034). The study was conducted in accordance with the principles in the Declaration of Helsinki [27] and the International Conference on Harmonisation Good Clinical Practice E6 (ICH-GCP), and in accordance with all applicable regulatory requirements. Written informed consent was obtained from all participants.

This trial was prospectively registered with the Chinese Clinical Trial Register (ChiCTR-IOR-16007700; 30th December 2015). The detailed protocol for this study has been published previously [28].

### Recruitment

**Fig 1:**
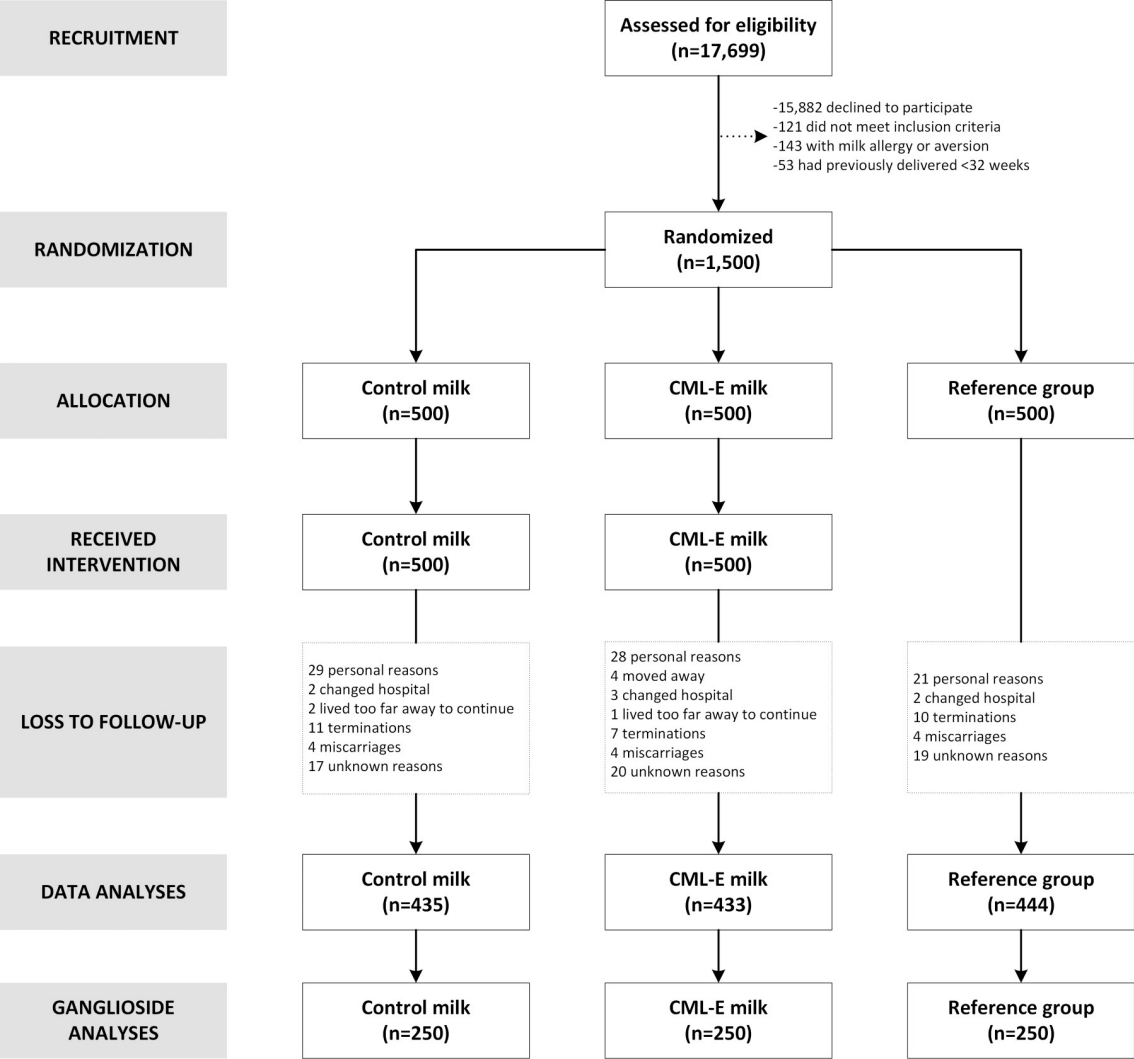
Flow of participants in the CLIMB trial (Chongqing, China).

Of 17,699 women screened for eligibility, a total of 1,500 were recruited from maternity clinics in the Chongqing municipality of China between 11 and 14 weeks of pregnancy in 2015–2017 (Fig 1). Eligible gravidas were aged 20 to 40 years and had a singleton pregnancy. Women with a history of premature delivery before 32 weeks of gestation, known milk allergy or aversion, or lactose intolerance were excluded. The maternity clinics were the Ranjiaba Branch and the Qixinggang branch of the Chongqing Health Centre for Women and Children, which is the major provider of maternity care in the region. These clinics are accredited as having the highest grade of care in Mainland China, and care for both high- and low-risk pregnant women.

### Intervention

Following recruitment, participants were randomly allocated to one of three treatment groups in a 1:1:1 ratio, using a computerised system as previously described [28]:

- Control Milk group–received a standard powdered maternal milk formulation (Fonterra Co-operative Group Limited, New Zealand) in 37.5g servings to be reconstituted with water and consumed twice a day, which provided a minimum of 4 mg gangliosides/day [28]. This milk formula was not enriched with additional complex milk lipids, so that the ganglioside dose consumed by participants in this group does not indicate supplementation, but instead the general effects of consuming milk.
- CML-enriched milk group (CML-E)–received a powdered maternal milk formulation with a minimum of 8 mg of gangliosides/day [28] and consumed as above. The CML-enriched maternal milk (Anmum™ maternal milk, Fonterra Co-operative Group Limited, New Zealand) contains an ingredient rich in MFGM as a source of CML (SureStart™ MFGM Lipid 100, Fonterra Co-operative Group Limited).

The major ganglioside present in both the control and treatment milk formulations was GD3 (>90% of total gangliosides). Both formulations were isocaloric, and had identical content of macronutrients and micronutrients, differing only in the concentration of MFGM (and therefore CMLs) [28]. Detailed nutritional information regarding both milk products is available in the published trial protocol [28]. Participants randomized to a milk formula received identical instructions for reconstitution and consumption. In brief, 37.5 g of powder was measured using the provided scoop, and added to 200 ml of water. The drink was then to be consumed in its entirety.

- Reference group–did not receive a milk product, but their obstetric care included prenatal folic acid supplementation of 400 mg/day (the same dose as the two groups that received milk supplements). They did not receive advice as to how much dairy they should consume.

Note that it was important to include the two treatment groups and a reference group in order to better tease out the likely cause of potential effects of the supplement. Milk formula is a complex intervention with many chemical constituents with potential biological effects. To determine the effect of consuming a milk supplement vs not consuming a milk supplement, a comparison could be made between the Control and Reference group. To determine the specific effects of increasing dietary complex milk lipid intake, a comparison could be made between the CML-E and Control milk groups.

All participants received standard pregnancy care through the First Affiliated Hospital of Chongqing Medical University.

### Blinding

Following randomised allocation, investigators, research nurses, and the participants themselves knew if they had been allocated to the Reference group (no intervention) or to a milk treatment group. However, where a participant was allocated to a milk product group, participants, research nurses, and investigators were blinded as to which milk product they received. This was maintained until after analyses on the primary outcome and pregnancy outcomes were completed. Milk product containers were labelled with a product number and colour code that enabled delivery to the correct participants while maintaining blinding.

### Assessments

Data were collected prospectively throughout the trial and women were assessed three times during pregnancy: at 11–14 weeks of gestation (baseline/visit 1), at 22–28 weeks (visit 2), and at 32–34 weeks (visit 3) at the First Affiliated Hospital of Chongqing Medical University. In addition, the mother and her baby were assessed at birth, 6 weeks, and 12 months after delivery. The details of assessments have been previously described [28]. They included maternal anthropometry, nutritional assessments, and blood sampling at all visits during pregnancy, as well as an oral glucose tolerance test at 22–28 weeks of gestation. Gestational diabetes was diagnosed based on the 75-g oral glucose tolerance test according to the IADPSG [29] and WHO criteria [30], defined by one of the following: fasting glucose ≥5.1 mmol/l, 1-hr glucose ≥10 mmol/l, or 2-hr glucose ≥8.5 mmol/l.

Pre-eclampsia was defined as systolic blood pressure ≥140 mmHg and/or diastolic blood pressure ≥90 mmHg on at least two occasions four hours apart after 20 weeks of gestation, with proteinuria (24-hour urinary protein ≥300 mg, spot urine protein:creatinine ratio ≥30 mg/mmol creatinine, or urine dipstick protein ≥2+). The diagnosis of all other complications of pregnancy was made by the participant’s health care providers. Babies were classified as small-for-gestational-age (SGA) and large-for-gestational-age (LGA) based on the 10^th^ and 90^th^ percentiles of birth weight for gestation, respectively, using recently developed birthweight references for Chongqing [31]. Compliance with treatment protocol was assessed by reviewing a log kept by each participant detailing when they consumed the milk products.

At visit 4, participants answered a food frequency questionnaire (FFQ) that reflected their dietary intake over the previous 3 months. This FFQ was an adaptation of the FFQ used in the S-PRESTO study in Singapore, to include food relevant to the Chongqing context [32]. This was used to determine the reported daily intake of liquid dairy products, which enabled evaluation of any overall effects of supplementation on dairy intake in comparison to the Reference group.

### Analysis of blood samples

Serum gangliosides and blood markers of nutritional status were analysed in a subset of 750 women (250 were randomly selected from each group). Women were eligible to be selected for detailed blood sample analyses if they had: 1) a baseline blood sample taken; 2) a second blood sample at 22–26 weeks of gestation; and 3) a third sample at 30–35 weeks.

Serum ganglioside concentrations were measured in the total lipid fraction by ultra-performance liquid chromatography-mass spectrometry (UPLC-MS). Standards for all gangliosides measured were purchased from Matreya Lipids and Biochemicals (PA, USA) and were used to produce 8-point calibration curves (0.78–10 μg/mL). The lipid fraction was extracted from 200 μL of serum by methanol/chloroform (2:1) extraction twice, according to a published protocol [33]. The gangliosides were isolated in the upper methanol phase, and separated using a Waters UPLC system (ACQUITY UPLC Class, Waters, Milford, MA) equipped with a ACQUITY BEH HILIC column (2.1 mm x 100 mm, 1.7 μm, Waters, Milford, MA) and BEH HILIC 1.7 μm Guard column (VanGuardTM, Waters, Milford, MA) operated at 45°C. Mobile phases A and B consisted of 95% acetonitrile with 0.1% formic acid (A), and 50% acetonitrile with 0.1% formic acid (B), respectively. The linear gradient was: 0 to 2 min, 1% B; 2 to 12.5 min, 1 to 95% B; 12.5 to 13 min, 95% B; 13 to 13.5, 95 to 1% B; 13.5 to 18 min, 1% B, with a flow rate of 0.4 ml/min. The autosampler was maintained at 4°C with an injection volume set to 5 μL. The mass spectrometer QTOF (XEVO G2-S) was operated in negative electrospray (ESI-) mode with a scan range from 500 to 1700 m/z, and a data scan rate of 0.4 s. The capillary voltage was set at -3000 V, with the ion source temperature at 120°C, while the cone gas and desolvation gas were set to 50 L/hr and 800 L/hr, respectively.

### Compliance

Participants were issued with a daily log to record when they used the milk powder they were allocated. These records were collected and used to calculate the percentage of milk drinks consumed, which was used as a measure of compliance.

### Study outcomes

The primary outcome was the total ganglioside concentration in maternal serum. Secondary outcomes included maternal nutritional status (e.g. micronutrient status and cell count in blood), clinical indicators of maternal health (e.g. blood pressure and glucose tolerance), gestational weight gain, pregnancy complications, as well as neonatal outcomes.

### Statistical methods

Comparisons of baseline characteristics among the subgroup of participants who had ganglioside levels measured were carried out using one-way ANOVA, Fisher’s exact tests, or non-parametric Kruskal-Wallis tests, as appropriate.

General linear mixed models with repeated measures were used to evaluate the treatment effects on the primary maternal outcome (i.e. total serum ganglioside levels) across pregnancy, using data collected at the two clinic visits after randomization as the outcomes. Models included participant ID as a random factor, randomization group and visit as fixed factors, also including as a covariate the value of the respective outcome at baseline. Model-adjusted group differences were estimated at each visit, by including an interaction term between treatment and visit. An unstructured covariance was adopted, chosen based on the best model fit statistics, although these parameters and the model results were virtually identical when run with other covariance structures (i.e. compound symmetry and first order autoregressive).

Potential differences in ganglioside levels in cord blood between groups were assessed using general linear regression models. These included as covariates the number of days elapsed between birth and the baseline visit (corresponding to the length of supplementation for the milk groups), as well as the maternal serum level of the respective ganglioside at baseline.

Maternal secondary outcomes with multiple measurements during pregnancy were examined as described for the primary outcome. Other continuous secondary outcomes were analysed using general linear regression models, adjusting for the baseline value if available. Note that, where appropriate, ganglioside and micronutrient data were log-transformed to approximate normality.

The associations between ganglioside levels in maternal serum at 32–34 weeks of gestation (last measurement) and cord blood were assessed using Pearson’s correlation coefficients.

Binary outcomes related to maternal health and pregnancy outcomes were analysed using generalized linear regression models, with pairwise relative risks obtained. As the number of doses prescribed and missed were recorded for the vast majority of participants, additional per-protocol analyses were carried out excluding participants who have taken less than 75% of prescribed milk doses.

Statistical analyses were conducted using SAS v9.4 (SAS Institute, Cary, North Carolina, USA) and SPSS v24 (IBM Corp, Armonk, NY, USA). All statistical tests were two-tailed and maintained at a 5% significance level, without adjustment for multiple comparisons. Where appropriate, outcome data are presented as model-adjusted means (estimated marginal means, back-transformed if necessary), with associated 95% confidence intervals.

### Power calculation

Our power calculation was based on the observed standard deviations of 0.07, 2.19, and 2.22 μg/mL for GD3, GM3, and total gangliosides, respectively. Our sample size of 250 women per group would be powered to detect statistically significant pairwise differences of 0.02 μg/mL (1.9%), 0.64 μg/mL (5.4%), and 0.64 μg/mL (5.0%), respectively, using a two-tailed t-test with 90% power and α = 0.05.

## Results

A total of 1,500 women were recruited into the study (Fig 1), with a mean age of 28.4 years (range 19–40 years). The vast majority of participants were of Han Chinese ethnicity (97.8%), nulliparous (78%), non-smokers at baseline assessment (99.9%), married or in a de facto relationship (99.8%), and had conceived without fertility treatment (98.7%). The baseline characteristics of participants in the three study groups are provided in Table 1.

**Table 1:** Baseline characteristics of the study population randomized into the CLIMB trial (Chongqing, China).

		Control milk	CML-E milk	Reference
**n**		500	500	500
**Age (years)**		28.4 ± 3.6	28.6 ± 3.5	28.3 ± 3.4
**Weight (kg)**		54.5 ± 7.7	54.5 ± 8.0	54.8 ± 8.5
**Height (cm)**		159.5 ± 4.5	159.6 ± 4.4	159.6 ± 4.6
**BMI (kg/m**^**2**^**)**		21.4 ± 2.9	21.4 ± 2.9	21.5 ± 3.1
**Underweight/normal weight (BMI <25 kg/m**^**2**^**)**		451 (90.2%)	444 (88.8%)	443 (88.6%)
**Overweight (BMI ≥25 and <30 kg/m**^**2**^**)**		43 (8.6%)	49 (9.8%)	49 (9.8%)
**Obesity (BMI ≥30 kg/m**^**2**^**)**		6 (1.2%)	7 (1.4%)	8 (1.6%)
**Gravidity**	**1**	232 (46.4%)	229 (45.8%)	235 (47%)
	**2**	141 (28.2%)	147 (29.4%)	138 (27.6%)
	**≥3**	127 (25.4%)	124 (24.8%)	127 (25.4%)
**Parity**	**0**	389 (77.8%)	398 (79.6%)	383 (76.6%)
	**1**	109 (21.8%)	101 (20.2%)	114 (22.8%)
	**2**	2 (0.4%)	1 (0.2%)	3 (0.6%)
**Fertility treatment**		11 (2.2%)	2 (0.4%)	6 (1.2%)
**Marital status (married or de facto)**		499 (99.8%)	499 (99.8%)	499 (99.8%)
**Years of schooling**		15.7 ± 1.8	15.6 ± 1.8	15.6 ± 1.9
**Tertiary education**		311 (62.2%)	322 (64.4%)	319 (63.8%)
**Ethnicity (Han Chinese)**		492 (98.4%)	482 (96.4%)	493 (98.6%)
**Tobacco smoking during pregnancy**		1 (0.2%)	nil	nil
**Alcohol consumption during pregnancy**		1 (0.2%)	nil	nil

Data are means ± standard deviations or n (%). BMI, body mass index.

CML-E represents the group of mothers who received milk enriched with complex milk lipids.

### Progression and compliance

Of 1,500 women who were randomized, 1,312 (87.5%) remained in the study so that their pregnancy outcomes were included in the analyses. Amongst those lost to follow-up, 12 miscarried, 28 had a termination of pregnancy, and 148 withdrew (with the majority of withdrawals before the second visit). Compliance with treatment was high, with 95% of participants consuming 75% or more of their prescribed milk product doses, and there were no differences in compliance between milk treatment groups [CML-E median = 88.8% (IQR = 9.2) vs Control milk 88.5% (IQR = 9.1); p = 0.40].

### Maternal serum and cord blood ganglioside levels

Across all groups, 860 participants were eligible for ganglioside analysis, and 250 women per group had serum ganglioside levels measured. There were no differences in baseline characteristics between those women who were randomly selected for blood analysis and those who were not (S1 Table). There were also no differences in baseline characteristics among the 3 subgroups with gangliosides measured (Table 2).

**Table 2:** Baseline characteristics of the study population randomized into the CLIMB trial (Chongqing, China), who were randomly selected to have ganglioside concentrations measured in maternal and cord blood.

		Control milk	CML-E milk	Reference	*P*-value
**n**		250	250	250	
**Age (years)**		28.5 ± 3.5	28.6 ± 3.3	28.1 ± 3.3	0.17
**Weight (kg)**		54.6 ± 8.0	54.4 ± 7.8	55.0 ± 8.0	0.65
**Height (cm)**		159.8 ± 4.9	159.7 ± 4.7	159.6 ± 4.6	0.88
**BMI (kg/m**^**2**^**)**		21.4 ± 3.1	21.3 ± 2.9	21.6 ± 2.8	0.61
**BMI status**					0.64
Underweight/normal weight (BMI <25 kg/m^2^)		228 (91.2%)	224 (89.6%)	220 (88.0%)	
Overweight (BMI ≥25 and <30 kg/m^2^)		18 (7.2%)	22 (8.8%)	28 (11.2%)	
Obesity (BMI ≥30 kg/m^2^)		4 (1.6%)	3 (1.2%)	3 (1.2%)	
**Gravidity**	**1**	127 (50.8%)	115 (46.0%)	114 (45.6%)	0.32
	**2**	72 (28.8%)	77 (30.8%)	67 (26.8%)	
	**≥3**	51 (20.4%)	57 (22.8%)	70 (28.0%)	
**Parity**	**0**	200 (80.0%)	198 (79.2%)	194 (77.6%)	0.84
	**1**	49 (19.6%)	51 (20.4%)	56 (22.4%)	
	**2**	1 (0.4%)	nil	1 (0.4%)	
**Fertility treatment**		2 (0.8%)	nil	5 (2.0%)	0.08
**Marital status (married or de facto)**		250 (100%)	250 (100%)	250 (100%)	>0.99
**Years of schooling**		15.8 ± 1.7	15.7 ± 1.8	15.6 ± 2.0	0.48
**Tertiary education**		82 (32.8%)	85 (34.0%)	91 (36.4%)	0.72
**Ethnicity (Han Chinese)**		246 (98.4%)	241 (96.4%)	247 (98.8%)	0.36
**Tobacco smoking during pregnancy**		nil	nil	nil	–
**Alcohol consumption during pregnancy**		nil	nil	nil	–

Data are means ± standard deviations or n (%), as appropriate.

*P*-values for continuous outcomes were derived from one-way ANOVA and those for categorical outcomes from Fisher’s exact tests.

BMI, body mass index.

CML-E represents the group of mothers who received milk enriched with complex milk lipids.

Mothers supplemented with CML-E milk had greater levels of GM3 (+2.8%; p = 0.034) and total gangliosides (+2.6%; p = 0.034) in serum compared with those who received Control milk, but were not different from the Reference group of unsupplemented women (Table 3). In contrast, the Control milk group had lower levels of GM3 (-2.9%; p = 0.023) and total gangliosides (-2.7%; p = 0.024) than the Reference group (Table 3).

**Table 3:** Concentrations of gangliosides in maternal serum and cord blood (μg/mL) according to treatment group in the CLIMB trial (Chongqing, China).

		Control milk	CML-E milk	Reference
**Maternal serum** ^**1**^	**n**	250	250	250
	**GD1a**	0.0207 (0.0204, 0.0210)	0.0210 (0.0207, 0.0213)	0.0210 (0.0207, 0.0213)
	**GD1b**	0.0192 (0.0188, 0.0195)	0.0194 (0.0191, 0.0198)	0.0196 (0.0193, 0.0200)
	**GD3**	1.039 (1.032, 1.045)	1.041 (1.034, 1.048)	1.041 (1.034, 1.048)
	**GM1**	0.0101 (0.0099, 0.0103)	0.0101 (0.0099, 0.0103)	0.0101 (0.0099, 0.0103)
	**GM2**	0.0401 (0.0396, 0.0406)	0.0401 (0.0396, 0.0406)	0.0401 (0.0395, 0.0406)
	**GM3**	11.55 (11.34, 11.76)	11.87 (11.66, 12.10)*	11.90 (11.69, 12.10)*
	**GT1b**	0.0099 (0.0098, 0.0101)	0.0101 (0.0099, 0.0102)	0.0102 (0.0100, 0.0103)
	**Total**	12.69 (12.48, 12.91)	13.02 (12.80, 13.23)*	13.04 (12.83, 13.25)*
**Cord blood** ^**2**^	**n**	76	61	80
	**GD1a**	0.141 (0.137, 0.145)	0.145 (0.140, 0.151)	0.142 (0.138, 0.147)
	**GD1b**	0.042 (0.039, 0.044)	0.040 (0.036, 0.043)	0.037 (0.034, 0.039)**
	**GD3**	0.600 (0.573, 0.627)	0.604 (0.569, 0.639)	0.594 (0.568, 0.639)
	**GM1**	0.611 (0.577, 0.646)	0.609 (0.564, 0.653)	0.623 (0.590, 0.656)
	**GM2**	0.100 (0.097, 0.103)	0.102 (0.098, 0.105)	0.102 (0.100, 0.105)
	**GM3**	8.55 (7.85, 9.26)	8.55 (7.63, 9.47)	8.32 (7.65, 8.99)
	**GT1b**	0.079 (0.076, 0.082)	0.081 (0.078, 0.085)	0.083 (0.080, 0.086)*
	**Total**	10.13 (9.40, 10.86)	10.12 (9.17, 11.08)	9.90 (9.205, 10.60)

Data are estimated marginal means and respective 95% confidence intervals.

*p<0.05 and

**p<0.01 vs Control milk.

^1^ Data were analysed using repeated measures adjusting for clinic visit, visit number vs group interaction, and the value of the respective outcome at baseline.

^2^ Data were adjusted for the number of days elapsed between birth and the baseline visit, as well as the maternal serum level of the respective ganglioside at baseline.

CML-E represents the group of mothers who received milk enriched with complex milk lipids.

There was no evidence that treatment effects between groups changed across pregnancy (S2 Table). Nonetheless, examination of the data for GM3 and total gangliosides per visit shows that the differences between groups were primarily observed in the third trimester of pregnancy (S3 Table).

Maternal supplementation with CML-E milk had no effect on ganglioside levels in cord blood when compared to the Reference group or Control milk group (Table 3). However, compared to the Reference group, control milk intake led to an increase in GD1b (+13.5%; p = 0.007) and a decrease in GT1b (-4.8%; p = 0.048) (which are minor gangliosides, recorded at low concentrations), but had no effect on any other gangliosides (Table 3). Of note, maternal ganglioside levels were not correlated with cord blood ganglioside levels (S4 Table).

### Pregnancy outcomes

CML-E milk supplementation had no effect on the risk of pregnancy loss (i.e. stillbirths or miscarriages), congenital abnormalities, delivery by caesarean section, or preterm delivery (Tables 4 and S5). In addition, the rate of gestational hypertension did not differ between groups (Table 4). However, both milk supplementation groups had marginally lower (≤1 mmHg) systolic blood pressure than the Reference group (Table 5), which is unlikely to be of any clinical significance.

**Table 4:** Rates of categorical pregnancy outcomes in the CLIMB trial (Chongqing, China) and the respective relative risks.

	Rates			Relative risks		
	Control milk	CML-E milk	Reference	CML-E milk vs Control milk	CML-E milk vs Reference	Control milk vs Reference
Pregnancy loss	4 (0.9%)	7 (1.6%)	6 (1.4%)	1.81 (0.53, 6.14)	1.20 (0.41, 3.53)	0.66 (0.19, 2.33)
Congenital abnormality	9 (2.0%)	4 (0.9%)	8 (1.8%)	0.47 (0.15, 2.84)	0.52 (0.16, 1.71)	1.11 (0.43, 2.84)
Induced labour	15 (3.5%)	15 (3.5%)	16 (3.7%)	1.02 (0.51, 2.07)	0.96 (0.48, 1.92)	0.94 (0.47, 1.87)
Caesarean delivery	211 (47.5%)	193 (45.1%)	216 (49.4%)	0.95 (0.82, 1.09)	1.09 (0.79, 1.05)	0.96 (0.84, 1.10)
Preterm delivery ^1^	22 (5.0%)	21 (4.9%)	27 (6.1%)	0.99 (0.55, 1.77)	0.80 (0.46, 1.40)	0.81 (0.47, 1.40)
Stillbirth	nil	3 (0.7%)	2 (0.5%)	–	–	–
Congenital abnormality	9 (2.0%)	4 (0.9%)	8 (1.8%)	0.47 (0.15, 1.51)	0.52 (0.16, 1.71)	1.11 (0.43, 2.84)
APGAR score <7 at 5 minutes	nil	nil	nil	–	–	–
SGA ^2^	29 (6.6%)	31 (7.4%)	27 (6.3%)	1.12 (0.69, 1.82)	1.17 (0.71, 1.93)	1.05 (0.63, 1.74)
LGA ^3^	39 (8.9%)	47 (11.2%)	37 (8.6%)	1.26 (0.84, 1.88)	1.30 (0.86, 1.95)	1.03 (0.67, 1.58)
Macrosomia ^4^	18 (4.1%)	29 (6.9%)	19 (4.5%)	1.68 (0.95, 2.99)	1.54 (0.88, 2.71)	0.92 (0.49, 1.72)
NICU admission ^5^	7 (1.6%)	10 (2.3%)	11 (2.5%)	1.49 (0.57, 3.87)	0.93 (0.40, 2.18)	0.63 (0.25, 1.61)
Gestational hypertension ^6^	9 (2.0%)	7 (1.6%)	4 (0.9%)	0.80 (0.31, 2.09)	2.04 (0.61, 6.82)	2.55 (0.80, 8.09)
Gestational diabetes ^7^	135 (30.8%)	106 (24.5%)	123 (27.6%)	0.80 (0.64, 0.99)*	0.89 (0.71, 1.11)	1.12 (0.91, 1.37)
Fasting glucose ≥5.1 mmol/l	72 (16.4%)	51 (11.8%)	81 (18.2%)	0.72 (0.51, 1.00)	0.65 (0.47, 0.90)**	0.90 (0.68, 1.21)
60-min glucose ≥10 mmol/l	56 (12.8%)	49 (11.3%)	49 (11.0%)	0.89 (0.62, 1.27)	1.03 (0.71, 1.49)	1.16 (0.81, 1.66)
120-min glucose ≥8.5 mmol/l	74 (16.9%)	55 (12.7%)	46 (10.3%)	0.75 (0.55, 1.04)	1.23 (0.85, 1.78)	1.63 (1.16, 2.30)**

Data are n (%) or relative risks and their 95% confidence intervals.

*p<0.05 and

**p<0.01 for the comparison between the two respective groups.

^1^ Birth at less than 37 weeks of gestation.

^2^ SGA = small-for-gestational-age (birth weight ≤10th percentile).

^3^ LGA = large-for-gestational-age (birth weight ≥90th percentile).

^4^ Macrosomia = birth weight ≥4.0 kg.

^5^ Neonatal intensive care unit.

^6^ Systolic blood pressure ≥140 mmHg and/or diastolic blood pressure ≥90 mmHg.

^7^ Measured during a 75-g oral glucose tolerance test at 22–28 weeks of gestation. Gestational diabetes defined by one of the following: fasting glucose ≥5.1mmol/l, 60-min glucose ≥10mmol/l, or 120-min glucose ≥8.5mmol/l.

CML-E represents the group of mothers who received milk enriched with complex milk lipids.

**Table 5:** Continuous pregnancy outcomes in the CLIMB trial (Chongqing, China).

	Control milk	CML-E milk	Reference
**Gestational weight gain (kg)** ^**1**^	8.96 (8.59, 9.33)	9.08 (8.71, 9.46)	8.69 (8.32, 9.06)
**Gestational age at delivery (days)**	276 (275, 277)	275 (274, 276)	275 (274, 276)
**Placental weight (g)**	562 (555, 570)	561 (553, 568)	553 (545, 560)
**Birth weight (g)**	3,322 (3,282, 3,363)	3,311 (3,270, 3,353)	3,292 (3,251, 3,333)
**Birth length (cm)**	49.8 (49.6, 49.9)	49.8 (49.6, 49.9)	49.7 (49.6, 49.9)
**Maternal SBP (mmHg)** ^**2**^	114.9 (114.3, 115.4)†	115.1 (114.5, 115.6)†	115.9 (115.3, 116.4)
**Maternal DBP (mmHg)** ^**2**^	70.6 (70.2, 71.0)	70.6 (70.2, 71.0)	70.7 (70.3, 71.1)

Data are estimated marginal means and respective 95% confidence intervals.

^†^*p*<0.05 for a comparison with the Reference group.

SBP, systolic blood pressure; DBP, diastolic blood pressure.

^1^ Measured between the baseline visit and the last visit during pregnancy at 32–34 weeks of gestation; means are adjusted for the respective number of days.

^2^ Values are derived from repeated measures analyses adjusting for clinic visit, visit number vs group interaction, and the value of the respective outcome at baseline.

CML-E represents the group of mothers who received milk enriched with complex milk lipids.

The overall risk of developing gestational diabetes with CML-E milk supplementation was similar to the Reference group, but lower than the risk with Control milk [relative risk (RR) 0.80; p = 0.039] (Table 4). Nonetheless, the risk of having an elevated fasting glucose that meets the diabetes threshold (≥5.1 mmol/l) was lower with CML-E milk compared to the Reference group (RR 0.65; p = 0.009) (Table 4). Conversely, control milk supplementation was associated with a 63% increase (i.e. RR 1.63; p = 0.005) in the risk of having an elevated 120-min glucose level meeting the diabetes threshold (≥8.5 mmol/l) compared to the Reference group (Table 4).

CML-E milk supplementation had no effect on gestational weight gain, gestational length, mean birth weight, birth length, or rates of SGA, LGA, or macrosomia in the offspring (Tables 4 & 5). There were no differences in the rate of NICU admission or any other neonatal complications (Table 4). Note that the rate of preeclampsia has not been reported as there was inconsistent measurement of urinary protein in those participants who developed elevated blood pressure, so that there were several cases where the diagnosis was not confirmed.

### Liquid dairy consumption

At visit 4, liquid dairy consumption (not including the trial supplement) was lower in the Control (176 ± 139 ml/day p<0.0001) and the CML-E (151 ± 126 ml/day; p<0.0001) groups in comparison to the Reference group (222 ± 131 ml/day). This indicates that the trial supplements of 400 ml/day milk formula led to a reduction in the consumption of other sources of dairy, but that when the supplement was accounted for, the treatment groups consumed substantially more dairy than the Reference group.

### Per protocol analyses

Per-protocol analyses were performed excluding the 5% of participants who consumed less than 75% of prescribed milk doses. However, results were unchanged and identical to those of the main analyses.

## Discussion

This large, three-group double-blind, randomized controlled trial aimed to determine the effects of consuming a CML-enriched milk supplement during pregnancy on maternal serum and fetal cord ganglioside levels, and to tease out the effects of the CML enrichment from the effects of consuming milk.

The CML-enriched milk supplement had no effects on maternal serum or fetal cord blood ganglioside levels when compared with women who did not receive a supplement, and also had no effects on the majority of pregnancy and neonatal outcomes. When the two milk groups were compared, the CML-E group had a lower rate of gestational diabetes, and higher levels of the ganglioside GM3, but whether these are true effects of ganglioside supplementation are unclear, as these parameters in the CML-E group were not different from the unsupplemented Reference group.

The effects of the supplemental milk products on ganglioside levels were subtle. While the CML-E group did have slightly greater levels of GM3 and total gangliosides than the Control milk group, they were not different from the Reference group who were not provided with a maternal milk supplement. In fact, the Control milk group had slightly lower levels of GM3 and total gangliosides than the Reference group. One possible interpretation could be that consuming the control milk formula led to a small reduction in serum gangliosides, and that this was prevented by consumption of a CML-E milk containing a higher concentration of gangliosides. However, there is no rationale or prior evidence to suggest that control milk would have a ganglioside-lowering effect or that such a small reduction may be of clinical significance. It is noteworthy that in a small previous study where non-pregnant adults were given a greater dose of CML than that used in our trial, there was an associated increase in the ganglioside GD3 [18]. This strongly suggests that gangliosides are indeed absorbed and incorporated into circulating lipid pools in human subjects. However, the absorption and metabolism of dietary gangliosides is not well understood, and the relationship between ganglioside consumption and serum levels may be complex. It is possible that the dose given in the present study might have been insufficient to increase serum levels above the non-supplemented Reference group. While women in the Reference group could have increased their intake of ganglioside-containing foods such as meat and dairy products as a result of knowing they were not consuming a supplement, and there was slightly greater consumption of liquid dairy products in the Reference group (+50–70 ml, when the trial supplement was not accounted for), it was of much lower volume than the 400 ml/day provided by the supplements.

Changes in the way gangliosides are distributed during pregnancy (between the mother and fetus, and among different tissues) could also account for the lack of difference in serum levels between groups and the minimal effects on cord blood levels. We observed no correlations between maternal serum and cord ganglioside levels. If gangliosides were preferentially transported across the placenta and deposited in the tissues known to have rapid uptake (such as the fetal brain), this could account for the lack of any correlations, and consequently could have prevented an increase in maternal serum levels with supplementation, even though tissue levels might have changed. In this case, such a potentially important outcome would be undetectable without tissue sample analysis.

There were inconsistent findings with regard to the effects of the CML-E and control maternal milk supplements on indices of glucose metabolism. While there was a lower rate of gestational diabetes in the CML-E group compared with Control, a protective effect of CML-E milk is unlikely, as the overall risk of gestational diabetes was not different between the CML-E and the Reference groups. In contrast, supplementation with the control maternal milk was associated with a greater overall risk of gestational diabetes than supplementation with CML-E milk. This was primarily due to a greater risk of an elevated 120-minute glucose in the Control milk group. Given that the control and enriched milks differed only in their enrichment with MFGM, two possible explanations for this could be advanced. First, consumption of a maternal milk during pregnancy could increase the risk of glucose intolerance, and the addition of complex milk lipids from MFGM to the milk formulation may ameliorate that effect. In support of this, the group receiving the CML-E milk did have a lower rate of elevated fasting glucose than the Reference group, suggesting the additional CMLs could have had a beneficial effect on glucose metabolism. Conversely, the elevated risk of gestational diabetes in the Control milk group could be a type 1 error. While the difference in 120-minute glucose was statistically significant with a relatively low p-value, this was a non-predicted finding in one of many secondary outcomes, and milk is not known to cause glucose intolerance. Gangliosides have not been demonstrated to have beneficial effects on glucose metabolism, and, in fact, higher cellular levels may interfere with insulin signaling [34–36]. Further, the increased gestational diabetes risk with the control milk was not accompanied by increased risks of any other complications known to be associated with diabetes, such as caesarean delivery, macrosomia, birth asphyxia, or neonatal hypoglycaemia. In this context, it seems more likely that the observed differences were spurious. However, future studies of ganglioside supplementation during pregnancy should assess the effects on glucose tolerance, insulin sensitivity, and gestational diabetes risk.

Women in the CML-E and Control milk groups received two servings of milk per day, but this did not lead to any difference in weight gain during pregnancy compared with the Reference group. This shows the treatments did not promote weight gain despite providing 1268 kJ/day. It is likely that this is because consuming the milk supplements led to minor reductions in the consumption of other foods and drinks. For example, we observed that both treatment groups reported lower liquid dairy consumption compared to the Reference group (when the supplement was excluded).

This study had important strengths, including the large size and highly homogenous population coming from a single large city in China. In addition, the 3-group design controlled for the potential effects of consuming maternal milk formula, so that the specific effects of the CML enrichment could be assessed. Lastly, a careful log of compliance was recorded. However, there were also limitations. First, for logistical reasons it was not possible to collect the used formula containers to further corroborate the compliance data. Second, as it was not possible to measure ganglioside levels in all participants, a potential bias could have been introduced, as, in principle, those participants with insufficient data could have been different to the remainder of the group. However, we contend that this is unlikely, as there were no differences in baseline characteristics between those who were and were not tested for gangliosides. Further, despite the reduced sample size for ganglioside analysis, the study was adequately powered to detect small differences in ganglioside concentrations between groups (90% power to detect a 5% difference). Lastly, because differences in the rate of gestational diabetes were not expected, specific tests of insulin secretion and insulin sensitivity were not conducted. Such tests might have helped to determine whether the observed differences reflected underlying changes in glucose metabolism. It is also important to consider that the rate of gestational diabetes seen in our participants (Reference group: 27.6%) was high, substantially greater than the recently estimated pooled prevalence in mainland China (14.8%) [37], and well above that reported in the United States (9.2%) [38]. While this was advantageous as it increased the power to detect potential differences between groups, it is possible that, if real, the effects of the milk supplements on glucose metabolism may not be as marked in populations who are at lower risk.

## Conclusions

Our study showed that maternal ganglioside supplementation during pregnancy using complex milk lipid enriched milk formula was not associated with any adverse health outcomes. This is important as emerging data suggest that there may be neurodevelopmental benefits of ganglioside supplementation in early life. While the MFGM supplementation did not increase serum ganglioside concentrations, the effects on tissue levels were not assessed, so direct effects on the fetus remain possible. There is a need for further research addressing the bioavailability and distribution of dietary gangliosides during pregnancy and across the life span. A follow-up study is underway to determine the effects of this treatment on infant neurodevelopment and growth.

## Supporting information

S1 Table Baseline characteristics for those participants who were included in the ganglioside analyses vs those not included. (PDF)

S2 Table *P*-values for the interaction term between randomization group and assessment visit during pregnancy, derived from repeated measures analyses comparing ganglioside levels in maternal serum. (PDF)

S3 Table Concentrations of total gangliosides and GM3 in maternal serum (μg/mL) according to treatment group in the CLIMB trial (Chongqing, China), at each visit during pregnancy, after supplementation was initiated. (PDF)

S4 Table Pearson’s correlation coefficients (r) and respective *p*-values for the linear associations between concentrations of gangliosides (μg/mL) in maternal serum at 32–34 weeks of gestation and cord blood (n = 135). (PDF)

S5 Table APGAR scores in newborns according to randomization group in the CLIMB trial (Chongqing, China). (PDF)

## References

[1] Yu RK, Nakatani Y, Yanagisawa M (2009) The role of glycosphingolipid metabolism in the developing brain. J Lipid Res 50: S440–S445. 10.1194/jlr.R800028-JLR200 18845618 PMC2674698

[2] Palmano K, Rowan A, Guillermo R, Guan J, McJarrow P (2015) The role of gangliosides in neurodevelopment. Nutrients 7: 3891–3913. 10.3390/nu7053891 26007338 PMC4446785

[3] McJarrow P, Schnell N, Jumpsen J, Clandinin T (2009) Influence of dietary gangliosides on neonatal brain development. Nutr Rev 67: 451–463. 10.1111/j.1753-4887.2009.00211.x 19674342

[4] Wang B, Brand-Miller J (2003) The role and potential of sialic acid in human nutrition. Eur J Clin Nutr 57: 1351–1369. 10.1038/sj.ejcn.1601704 14576748

[5] Tsai Y-T, Itokazu Y, Robert KY (2016) GM1 ganglioside is involved in epigenetic activation loci of neuronal cells. Neurochem Res 41: 107–115. 10.1007/s11064-015-1742-7 26498762 PMC4775412

[6] Wang Q, Song Y, Tang Z, Wang Z, Xu Q, Bao N (2016) Effects of ganglioside GM1 and neural growth factor on neural stem cell proliferation and differentiation. Genet Mol Res 15: gmr.15038376. 10.4238/gmr.15038376 27525911

[7] Koon NA, Itokazu Y, Yu RK (2015) Ganglioside-dependent neural stem cell proliferation in Alzheimer’s disease model mice. ASN Neuro 7: 1759091415618916 10.1177/1759091415618916 26699276 PMC4710121

[8] Wang J, Cheng A, Wakade C, Robert KY (2014) Ganglioside GD3 is required for neurogenesis and long-term maintenance of neural stem cells in the postnatal mouse brain. J Neurosci 34: 13790–13800. 10.1523/JNEUROSCI.2275-14.2014 25297105 PMC4188974

[9] Jeon Y, Kim B, Kim JE, Kim BR, Ban S, Jeong JH, et al (2016) Effects of ganglioside on working memory and the default mode network in individuals with subjective cognitive impairment: a randomized controlled trial. Am J Chin Med 44: 489–514. 10.1142/S0192415X16500270 27109158

[10] Jung WR, Kim HG, Kim KL (2008) Ganglioside GQ1b improves spatial learning and memory of rats as measured by the Y-maze and the Morris water maze tests. Neurosci Lett 439: 220–225. 10.1016/j.neulet.2008.05.020 18514410

[11] Guan J, MacGibbon A, Zhang R, Elliffe DM, Moon S, Liu D-X (2015) Supplementation of complex milk lipid concentrate (CMLc) improved the memory of aged rats. Nutr Neurosci 18: 22–29. 10.1179/1476830513Y.0000000096 24257209

[12] Liu H, Radlowski EC, Conrad MS, Li Y, Dilger RN, Johnson RW (2014) Early supplementation of phospholipids and gangliosides affects brain and cognitive development in neonatal piglets. J Nutr 144: 1903–1909. 10.3945/jn.114.199828 25411030 PMC4230208

[13] Vickers MH, Guan J, Gustavsson M, Krägeloh CU, Breier BH, Davison M, et al (2009) Supplementation with a mixture of complex lipids derived from milk to growing rats results in improvements in parameters related to growth and cognition. Nutr Res 29: 426–435. 10.1016/j.nutres.2009.06.001 19628110

[14] Gurnida DA, Rowan AM, Idjradinata P, Muchtadi D, Sekarwana N (2012) Association of complex lipids containing gangliosides with cognitive development of 6-month-old infants. Early Hum Dev 88: 595–601. 10.1016/j.earlhumdev.2012.01.003 22289412

[15] Timby N, Domellöf E, Hernell O, Lönnerdal B, Domellöf M (2014) Neurodevelopment, nutrition, and growth until 12 mo of age in infants fed a low-energy, low-protein formula supplemented with bovine milk fat globule membranes: a randomized controlled trial. Am J Clin Nutr 99: 860–868. 10.3945/ajcn.113.064295 24500150

[16] Svennerholm L, Vanier MT (1972) The distribution of lipids in the human nervous system. II. Lipid composition of human fetal and infant brain. Brain Res 47: 457–468. 10.1016/0006-8993(72)90652-x 4642572

[17] Vanier MT, Holm M, Öhman R, Svennerholm L (1971) Developmental profiles of gangliosides in human and rat brain. J Neurochem 18: 581–592. 10.1111/j.1471-4159.1971.tb11988.x 5581573

[18] Miklavcic JJ, Shoemaker GK, Schnabl KL, Larsen BMK, Thomson ABR, Mazurak VC, et al (2017) Ganglioside intake increases plasma ganglioside content in human participants. J Parenter Enteral Nutr 41: 657–666. 10.1177/0148607115620093 26673692

[19] Hungund B, Morishima H, Gokhale V, Cooper T (1993) Placental transfer of (3H)-GM1 and its distribution to maternal and fetal tissues of the rat. Life Sci 53: 113–119. 10.1016/0024-3205(93)90658-p 8515685

[20] Mitchell M, Henare K, Balakrishnan B, Lowe E, Fong B, McJarrow P (2012) Transfer of gangliosides across the human placenta. Placenta 33: 312–316. 10.1016/j.placenta.2011.12.018 22225907

[21] Gustavsson M, Hodgkinson SC, Fong B, Norris C, Guan J, Krageloh CU, et al (2010) Maternal supplementation with a complex milk lipid mixture during pregnancy and lactation alters neonatal brain lipid composition but lacks effect on cognitive function in rats. Nutr Res 30: 279–289. 10.1016/j.nutres.2010.04.005 20534331

[22] Vesper H, Schmelz EM, Nikolova-Karakashian MN, Dillehay DL, Lynch DV, Merrill AH Jr., (1999) Sphingolipids in food and the emerging importance of sphingolipids to nutrition. J Nutr 129: 1239–1250. 10.1093/jn/129.7.1239 10395583

[23] Singh H, Gallier S (2017) Nature’s complex emulsion: the fat globules of milk. Food Hydrocolloids 68: 81–89.

[24] Bourlieu C, Michalski M-C (2015) Structure–function relationship of the milk fat globule. Curr Opin Clin Nutr Metab Care 18: 118–127. 10.1097/MCO.0000000000000138 25581036

[25] Ma L, Liu X, MacGibbon AK, Rowan A, McJarrow P, Fong BY (2015) Lactational changes in concentration and distribution of ganglioside molecular species in human breast milk from Chinese mothers. Lipids 50: 1145–1154. 10.1007/s11745-015-4073-1 26404454

[26] Giuffrida F, Cruz‐Hernandez C, Flück B, Tavazzi I, Thakkar SK, Destaillats F, et al (2013) Quantification of phospholipids classes in human milk. Lipids 48: 1051–1058. 10.1007/s11745-013-3825-z 23982210 PMC3779592

[27] World Medical Association (2013) World Medical Association Declaration of Helsinki: ethical principles for medical research involving human subjects. JAMA 310: 2191–2194. 10.1001/jama.2013.281053 24141714

[28] Huang S, Mo T-T, Norris T, Sun S, Zhang T, Han T-L, et al (2017) The CLIMB (Complex Lipids In Mothers and Babies) study: protocol for a multicentre, three-group, parallel randomised controlled trial to investigate the effect of supplementation of complex lipids in pregnancy, on maternal ganglioside status and subsequent cognitive outcomes in the offspring. BMJ Open 7: e016637 10.1136/bmjopen-2017-016637 29025835 PMC5652542

[29] Lapolla A, Dalfrà M, Ragazzi E, De Cata A, Fedele D (2011) New International Association of the Diabetes and Pregnancy Study Groups (IADPSG) recommendations for diagnosing gestational diabetes compared with former criteria: a retrospective study on pregnancy outcome. Diabet Med 28: 1074–1077. 10.1111/j.1464-5491.2011.03351.x 21658125

[30] World Health Organization (2013) Diagnostic criteria and classification of hyperglycaemia first detected in pregnancy. https://apps.who.int/iris/handle/10665/85975. 24199271

[31] Zhao X, Xia Y, Zhang H, Baker PN, Norris T (2019) Birth weight charts for a Chinese population: an observational study of routine newborn weight data from Chongqing. BMC Pediatr 19: 426 10.1186/s12887-019-1816-9 31711440 PMC6844044

[32] Loy SL, Cheung YB, Soh SE, Ng S, Tint MT, Aris IM, et al (2018) Female adiposity and time-to-pregnancy: a multiethnic prospective cohort. Hum Reprod 33: 2141–2149. 10.1093/humrep/dey300 30285230 PMC6201836

[33] Fong B, Norris C, Lowe E, McJarrow P (2009) Liquid chromatography-high-resolution mass spectrometry for quantitative analysis of gangliosides. Lipids 44: 867–874. 10.1007/s11745-009-3327-1 19633991

[34] Yamashita T, Hashiramoto A, Haluzik M, Mizukami H, Beck S, Norton A, et al (2003) Enhanced insulin sensitivity in mice lacking ganglioside GM3. PNAS 100: 3445–3449. 10.1073/pnas.0635898100 12629211 PMC152312

[35] Lipina C, Hundal HS (2015) Ganglioside GM3 as a gatekeeper of obesity-associated insulin resistance: evidence and mechanisms. FEBS Lett 589: 3221–3227. 10.1016/j.febslet.2015.09.018 26434718

[36] Sasaki N, Itakura Y, Toyoda M (2015) Ganglioside GM1 contributes to the state of insulin resistance in senescent human arterial endothelial cells. J Biol Chem 290: 25475–25486. 10.1074/jbc.M115.684274 26338710 PMC4646194

[37] Gao C, Sun X, Lu L, Liu F, Yuan J (2019) Prevalence of gestational diabetes mellitus in mainland China: a systematic review and meta-analysis. J Diabetes Investig 10: 154–162. 10.1111/jdi.12854 29683557 PMC6319492

[38] DeSisto CL, Kim SY, Sharma AJ (2014) Prevalence estimates of gestational diabetes mellitus in the United States, pregnancy risk assessment monitoring system (PRAMS), 2007–2010. Prev Chronic Dis 11: E104 10.5888/pcd11.130415 24945238 PMC4068111

