# Supplementary material for: Supplementation with milk enriched with complex lipids during pregnancy: A double-blind randomized controlled trial
Source: PLoS One. 2021 Feb 24;16(2):e0244916. doi: 10.1371/journal.pone.0244916 (PMC7904220; doi:10.1371/journal.pone.0244916)
Supplement: S1 Table — (PDF) [file pone.0244916.s001.pdf]

Table S1

Baseline characteristics for those participants who were included in the ganglioside analyses vs those not included.

		Included	Not included	*P*-value
**n**		750	750	
**Age (years)**		28.4 ± 3.4	28.5 ± 3.6	0.70
**Weight (kg)**		54.7 ± 7.9	54.5 ± 8.2	0.75
**Height (cm)**		159.7 ± 4.7	159.4 ± 4.3	0.17
**BMI (kg/m²)**		21.4 ± 2.9	21.4 ± 3.0	0.84
**Underweight/normal weight (BMI <25 kg/m²)**		579 (77.2%)	574 (76.5%)	0.97
**Overweight (BMI ≥25 and <30 kg/m²)**		68 (9.1%)	73 (9.7%)	
**Obesity (BMI ≥30 kg/m²)**		10 (1.3%)	11 (1.5%)	
**Gravidity**	**1**	356 (47.5%)	340 (45.3%)	0.22
	**2**	216 (28.8%)	210 (28.0%)	
	**≥3**	178 (23.7%)	200 (26.7%)	
**Fertility treatment**		7 (0.9%)	12 (1.6%)	0.13
**Marital status (married or de facto)**		749 (99.9%)	748 (99.7%)	0.56
**Years of schooling**		15.7 ± 1.8	15.6 ± 1.8	0.06
**Tertiary education**		490 (65.3%)	455 (60.7%)	0.11
**Ethnicity (Han Chinese)**		734 (97.9%)	733 (97.7%)	0.86

Data are means ± standard deviations or n (%), as appropriate.
BMI, body mass index.
P-values were derived from one-way ANOVA or chi-squared tests, as appropriate.
