# Supplementary material for: Supplementation with milk enriched with complex lipids during pregnancy: A double-blind randomized controlled trial
Source: PLoS One. 2021 Feb 24;16(2):e0244916. doi: 10.1371/journal.pone.0244916 (PMC7904220; doi:10.1371/journal.pone.0244916)
Supplement: S2 Table — (PDF) [file pone.0244916.s002.pdf]

Table S2

***P*-values for the interaction term between randomization group and assessment visit during pregnancy, derived from repeated measures analyses comparing ganglioside levels in maternal serum.**

	**Group * visit**
**GD1a**	0.75
**GD1b**	0.97
**GD3**	0.23
**GM1**	0.79
**GM2**	0.11
**GM3**	0.35
**GT1b**	0.77
**Total gangliosides**	0.33
