# Supplementary material for: Supplementation with milk enriched with complex lipids during pregnancy: A double-blind randomized controlled trial
Source: PLoS One. 2021 Feb 24;16(2):e0244916. doi: 10.1371/journal.pone.0244916 (PMC7904220; doi:10.1371/journal.pone.0244916)
Supplement: S3 Table — (PDF) [file pone.0244916.s003.pdf]

Table S3

Concentrations of total gangliosides and GM3 in maternal serum (μg/mL) according to treatment group in the CLIMB trial (Chongqing, China), at each visit during pregnancy, after supplementation was initiated.

CML-E represents the group of mothers who received milk enriched with complex milk lipids.

	Weeks of gestation	Control milk	CML-E milk	Reference	CML-E vs Control	CML-E vs Reference	Control vs Reference
**GM3**	**22–28**	11.68 (11.42, 11.93)	11.84 (11.58, 12.10)	11.92 (11.66, 12.17)	0.17 (-0.20, 0.53)	-0.07 (-0.43, 0.29)	-0.24 (-0.60, 0.12)
					p=0.36	p=0.70	p=0.20
	**32–34**	11.43 (11.17, 11.69)	11.90 (11.65, 12.16)	11.88 (11.62, 12.13)	**0.47 (0.11, 0.84)**	0.03 (-0.34, 0.39)	**-0.45 (-0.81, -0.08)**
					**p=0.011**	p=0.88	**p=0.017**
**Total gangliosides**	**22–28**	12.82 (12.56, 13.08)	12.99 (12.72, 13.25)	13.06 (12.80, 13.32)	0.17 (-0.20, 0.53)	-0.07 (-0.44, 0.29)	-0.24 (-0.61, 0.13)
					p=0.38	p=0.70	p=0.20
	**32–34**	12.57 (12.30, 12.83)	13.05 (12.79, 13.31)	13.02 (12.76, 13.28)	**0.48 (0.11, 0.85)**	0.03 (-0.34, 0.40)	**-0.45 (-0.82, -0.08)**
					**p=0.011**	p=0.87	**p=0.016**

Data are adjusted means or adjusted mean differences and respective 95% confidence intervals (CI), derived from repeated measures analyses, with the *p*-values for pairwise comparisons also provided.
P-values for statistically significant differences (at *p*<0.05) are shown in bold.
