# Supplementary material for: Supplementation with milk enriched with complex lipids during pregnancy: A double-blind randomized controlled trial
Source: PLoS One. 2021 Feb 24;16(2):e0244916. doi: 10.1371/journal.pone.0244916 (PMC7904220; doi:10.1371/journal.pone.0244916)
Supplement: S4 Table — (PDF) [file pone.0244916.s004.pdf]

Table S4

Pearson's correlation coefficients (r) and respective *p*-values for the linear associations between concentrations of gangliosides (µg/mL) in maternal serum at 32–34 weeks of gestation and cord blood (n=135).

	r	*P*-value
**GD1a**	0.04	0.69
**GD1b**	0.02	0.79
**GD3**	-0.05	0.54
**GM1**	-0.03	0.69
**GM2**	0.00	0.96
**GM3**	0.04	0.67
**GT1b**	0.00	0.97
**Total gangliosides**	0.05	0.54
