# Supplementary material for: Supplementation with milk enriched with complex lipids during pregnancy: A double-blind randomized controlled trial
Source: PLoS One. 2021 Feb 24;16(2):e0244916. doi: 10.1371/journal.pone.0244916 (PMC7904220; doi:10.1371/journal.pone.0244916)
Supplement: S5 Table — (PDF) [file pone.0244916.s005.pdf]

Table S5

APGAR scores in newborns according to randomization group in the CLIMB trial (Chongqing, China).

CML-E represents the group of mothers who received milk enriched with complex milk lipids.

	Control milk	CML-E milk	Reference	*P*-value
**n**	250	250	250	
**APGAR at 1 minute**	10 [9, 10]	10 [9, 10]	10 [9, 10]	0.93
**APGAR at 5 minutes**	10 [10, 10]	10 [10, 10]	10 [10, 10]	0.97

Data are medians [quartile 1, quartile 3].
P-values were derived from non-parametric Kruskal-Wallis tests, adjusted for ties.
